# Cognitive Activity and Functional Brain Networks — A Randomized Controlled Trial

**DOI:** 10.1002/alz70860_099748

**Published:** 2025-12-23

**Authors:** Allen Ting‐chun Lee, Yishan Luo, Zhaohua Huo, Lin Shi, Winnie Chiu Wing Chu, Linda Chiu‐wa Lam

**Affiliations:** ^1^ The Chinese University of Hong Kong, New Territories, Hong Kong SAR, China; ^2^ BrainNow Research Institute, Hong Kong Science and Technology Park, New Territories, Hong Kong, China; ^3^ The Chinese University of Hong Kong, New Territories, Hong Kong, China

## Abstract

**Background:**

Building on our previous findings that increased participation in cognitive activities, such as calligraphy practice, improves working memory and strengthens functional connectivities within the Default Mode Network in older adults with Subjective Cognitive Decline, we conducted a secondary analysis to examine the effects on other functional brain networks.

**Method:**

A total of 112 participants (aged 55‐75 years, CDR 0 but with Subjective Cognitive Decline, practicing ≥1 hour of calligraphy per week) were randomly assigned to either the control group (continuing usual calligraphy practice) or the intervention group (doubling the practice time for 6 months) in a 1:1 ratio. Functional connectivities within the Salience Network and Central Executive Network were assessed using functional MRI before and after the intervention. This assessor‐blind randomized controlled trial was funded by the Research Grants Council in Hong Kong (grant number: 24114519).

**Result:**

The intervention group showed stronger functional connectivities between core components of the Salience Network, including the anterior insula, supramarginal gyrus, and prefrontal cortex, over the 6‐month period, whereas the control group did not. No significant group differences were observed in the changes of functional connectivities within the Central Executive Network.

**Conclusion:**

Our findings suggest that increasing participation enhances the ability of the Salience Network to integrate and process salient stimuli, potentially contributing to improved cognitive regulation. Engaging more in cognitive activities may serve as a safe and effective non‐pharmacological intervention for maintaining cognitive function and network integrity in older adults. Future research should explore the long‐term benefits and potential mechanisms underlying these network‐specific changes.

